# What is the Healthy Gut Microbiota Composition? A Changing Ecosystem across Age, Environment, Diet, and Diseases

**DOI:** 10.3390/microorganisms7010014

**Published:** 2019-01-10

**Authors:** Emanuele Rinninella, Pauline Raoul, Marco Cintoni, Francesco Franceschi, Giacinto Abele Donato Miggiano, Antonio Gasbarrini, Maria Cristina Mele

**Affiliations:** 1 UOC di Nutrizione Clinica, Dipartimento di Scienze Gastroenterologiche, Endocrino-Metaboliche e Nefro-Urologiche, Fondazione Policlinico Universitario A. Gemelli IRCCS, 00168 Rome, Italy; giacintoabele.miggiano@unicatt.it (G.A.D.M.); mariacristina.mele@unicatt.it (M.C.M.); 2 Istituto di Patologia Speciale Medica, Università Cattolica del Sacro Cuore, 00168 Rome, Italy; pauline.raoul1@gmail.com (P.R.); antonio.gasbarrini@unicatt.it (A.G.); 3 Scuola di Specializzazione in Scienza dell’Alimentazione, Università di Roma Tor Vergata, 00133 Rome, Italy; marco.cintoni@gmail.com; 4 UOC di Medicina d’Urgenza e Pronto Soccorso, Dipartimento di Scienze dell’Emergenza, Anestesiologiche e della Rianimazione, Fondazione Policlinico Universitario A. Gemelli IRCCS, 00168 Rome, Italy; francesco.franceschi@unicatt.it; 5 Istituto di Medicina Interna e Geriatria, Università Cattolica del Sacro Cuore, 00168 Rome, Italy; 6 UOC di Medicina Interna e Gastroenterologia, Dipartimento di Scienze Gastroenterologiche, Endocrino-Metaboliche e Nefro-Urologiche, Fondazione Policlinico Universitario A. Gemelli IRCCS, 00168 Rome, Italy

**Keywords:** gut microbiota, diversity, health, diet, nutrition, age, milk feeding, necrotizing enterocolitis, weaning, enterotypes, irritable bowel syndrome, inflammatory bowel disease, celiac disease, colorectal cancer, obesity, type 2 diabetes, Alzheimer’s disease, Parkinson’s disease, hepatic encephalopathy, autism spectrum disorders, personalized medicine

## Abstract

Each individual is provided with a unique gut microbiota profile that plays many specific functions in host nutrient metabolism, maintenance of structural integrity of the gut mucosal barrier, immunomodulation, and protection against pathogens. Gut microbiota are composed of different bacteria species taxonomically classified by genus, family, order, and phyla. Each human’s gut microbiota are shaped in early life as their composition depends on infant transitions (birth gestational date, type of delivery, methods of milk feeding, weaning period) and external factors such as antibiotic use. These personal and healthy core native microbiota remain relatively stable in adulthood but differ between individuals due to enterotypes, body mass index (BMI) level, exercise frequency, lifestyle, and cultural and dietary habits. Accordingly, there is not a unique optimal gut microbiota composition since it is different for each individual. However, a healthy host–microorganism balance must be respected in order to optimally perform metabolic and immune functions and prevent disease development. This review will provide an overview of the studies that focus on gut microbiota balances in the same individual and between individuals and highlight the close mutualistic relationship between gut microbiota variations and diseases. Indeed, dysbiosis of gut microbiota is associated not only with intestinal disorders but also with numerous extra-intestinal diseases such as metabolic and neurological disorders. Understanding the cause or consequence of these gut microbiota balances in health and disease and how to maintain or restore a healthy gut microbiota composition should be useful in developing promising therapeutic interventions.

## 1. Introduction

The human gastrointestinal (GI) tract contains an abundant and diverse microbial community that gathers more than 100 trillion microorganisms [1]. The density of bacterial cells in the colon has been estimated at 10^11^ to 10^12^ per milliliter which makes the colon one of the most densely populated microbial habitats known on earth [2]. The gut microbiome encodes over 3 million genes producing thousands of metabolites, whereas the human genome consists of approximately 23,000 genes [3]. For years, scientists have been interested in gut microbiota, but one of the major difficulties in the relevant research has been the ability to culture these microorganisms [4]. During the last years, new technologies have allowed researchers to phylogenetically identify and/or quantify the components of the gut microbiota by analyzing nucleic acids (DNA and RNA) directly extracted from stools. The majority of these techniques are based on the extraction of DNA and the amplification of the 16S ribosomal RNA gene (rRNA) [5,6]. 16S rRNA sequencing has become the most useful technique to highlight diversity and abundance of the microbiome. The 16S rRNA gene sequences can be exploited with polymerase chain reaction (PCR) and metagenomics sequencing to characterize the microbial strains [7].

Considering the characteristics of gut microbiota such as the large diversity, the stability and resilience, and the symbiotic interaction with the host, we can define the host and the microorganisms inhabiting it as a “superorganism” [8,9] which performs immune and metabolic functions [1]. Gut bacteria are key regulators of digestion along the gastrointestinal tract; commensal bacteria play an important role in the extraction, synthesis, and absorption of many nutrients and metabolites, including bile acids, lipids, amino acids, vitamins, and short-chain fatty acids (SCFAs). Gut microbiota have a crucial immune function against pathogenic bacteria colonization inhibiting their growth, consuming available nutrients and/or producing bacteriocins. Gut microbiota also prevent bacteria invasion by maintaining the intestinal epithelium integrity [10]. Microorganisms prevent pathogenic colonization by many competition processes: nutrient metabolism, pH modification, antimicrobial peptide secretions, and effects on cell signaling pathways. Moreover, recent studies have identified a critical role for commensal bacteria and their products in regulating the development, homeostasis, and function of innate and adaptive immune cells [11]. It is paradoxical to note that the gut microbiota functions are highly preserved between individuals, whereas each individual’s gut microbiota are characterized by a specific combination of bacterial species due to inter-individual and intra-individual variations throughout human life.

This review aims to define what would be the optimal gut microbiota composition in order to maintain these optimal microbiota immune and metabolic functions. After defining microbiota variations within and between individuals, we highlight the involvement of gut microbiota dysbiosis in various intestinal and extra-intestinal disorder development and we describe the strong relationship between gut microbiota diversity and health. We will see how these gut microbiota variations may have huge implications for intestinal and extra-intestinal disorders and, consequently, may influence health.

## 2. Gut Microbiota Variations

**Figure 1:**
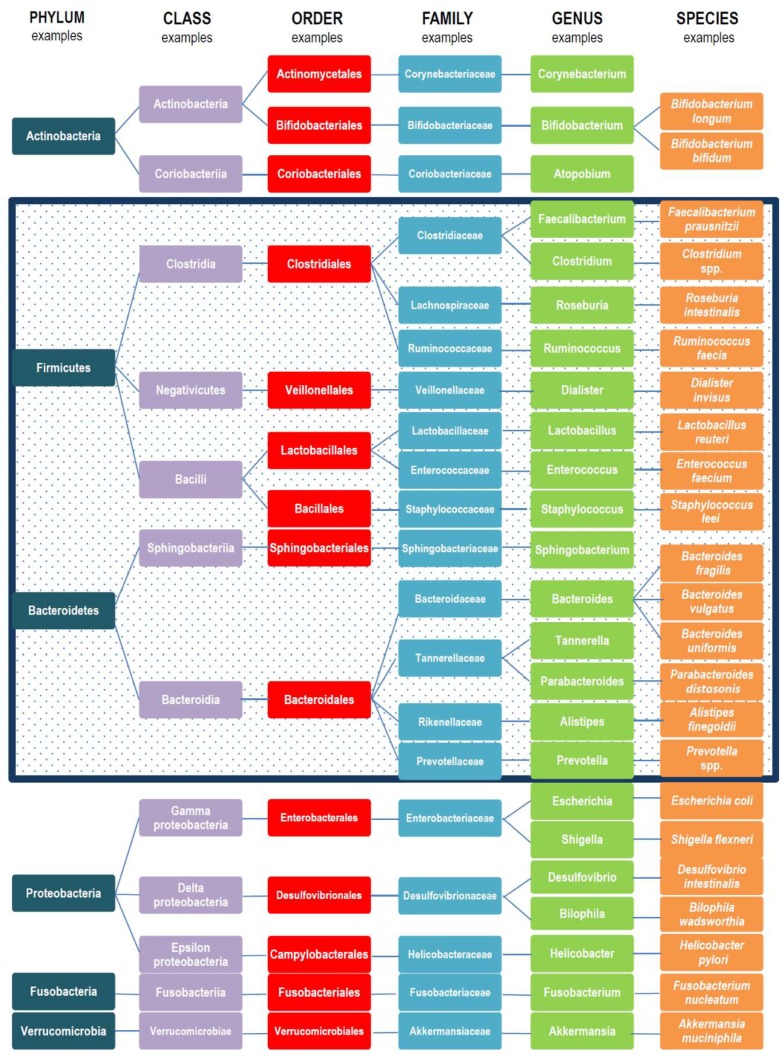
Examples of taxonomic gut microbiota composition. In the box are cited examples of bacteria belonging to Phyla Firmicutes and Bacteroidetes, representing 90% of gut microbiota.

Gut microbiota are composed of several species of microorganisms, including bacteria, yeast, and viruses. Taxonomically, bacteria are classified according to phyla, classes, orders, families, genera, and species. Only a few phyla are represented, accounting for more than 160 species [12]. The dominant gut microbial phyla are Firmicutes, Bacteroidetes, Actinobacteria, Proteobacteria, Fusobacteria, and Verrucomicrobia, with the two phyla Firmicutes and Bacteroidetes [13] representing 90% of gut microbiota. The Firmicutes phylum is composed of more than 200 different genera such as *Lactobacillus*, *Bacillus*, *Clostridium*, *Enterococcus,* and *Ruminicoccus*. *Clostridium* genera represent 95% of the Firmicutes phyla. Bacteroidetes consists of predominant genera such as *Bacteroides* and *Prevotella*. The Actinobacteria phylum is proportionally less abundant and mainly represented by the *Bifidobacterium* genus [13]. Examples of taxonomic gut microbiota composition are illustrated in Figure 1.

### 2.1. Variations in the Same Individual

Human gut microbiota vary taxonomically and functionally in each part of the GI tract and undergo variations in the same individual due to infant transitions, age, and environmental factors such as antibiotic use. Microbiota variations within individuals are summarized in Table 1.

#### 2.1.1. Intestine Anatomical Regions

Gut microbiota vary according to the intestine anatomical regions, which vary in terms of physiology, pH and O_2_ tension, digesta flow rates (rapid in the mouth to the caecum, slower afterward), substrate availability, and host secretions [14]. The small intestine provides a more challenging environment for microbial colonizers given the fairly short transit times (3–5 h) and the high bile concentrations. The large intestine, which is characterized by slow flow rates and neutral to mildly acidic pH, harbors by far the largest microbial community (dominated by obligate anaerobic bacteria) [14]. We can observe a microbiota quantitative increasing gradient and a microbiota qualitative decreasing gradient with a progressive aerobic bacteria decrease for the benefit of strictly anaerobic bacteria (Table 1).

#### 2.1.2. Infant Transitions

##### Birth Gestational Age

Birth gestational age is a major determinant of gut microbiota colonization. The microbiota composition of preterm infants (<37 weeks of gestation) is different from term counterparts (Table 1). In preterm infants, after birth, the microbiota colonization is challenged by organ immaturity and environmental factors such as antibiotic use, hospital stay [15], and enteral feeding [16]. For these reasons, the postnatal maturation of gut and systemic immunity may be highly affected by preterm birth [16].

In a new-born pig model, an analysis of the gut microbiota composition of preterm and term pigs showed that *Ruminococcus* spp., some *Enterobacterium* spp., *Lachnospiraceae*, *Peptostreptococcaceae*, and *Clostridiaceae* were dominant genera in both preterm and term pigs. However, a higher *Enterococcus* spp. abundance in preterm than term pigs was found [16].

Preterm infants show low diversity with an increased colonization of potentially pathogenic bacteria from the *Enterobacteriaceae* family of the Proteobacteria phylum [15] and reduced levels of strict anaerobes such as *Bifidobacterium* [17], *Bacteroides*, and *Atopobium* [15].

Human milk composition depends on genetic factors and the mother’s secretor and Lewis blood groups defining four phenotypes characterized by different levels of oligosaccharide amounts [18]. Higher levels of Proteobacteria and lower levels of Firmicutes are noted in premature infants of non-secretor mothers [19]. A recent study by Praticò et al. [20] characterizing human breast milk composition demonstrated that human milk oligosaccharides (HMOs) related to different mother phenotypes modulate gut microbiota composition in infants. Specifically, HMOs associated with secretor mothers may have a protective prebiotic effect by decreasing pathogens associated with sepsis and necrotizing enterocolitis (NEC) [19]. This indicates that HMOs may influence the intestinal microbiota and prevent gut dysfunction and NEC in preterm infants [21].

Lactoferrin (LF) is another well-known component of human milk favoring gut infant colonization with beneficial bacteria and consequently representing a crucial role played in preterm infants’ microbiota [22]. Indeed, correlations between LF and beneficial microbiota in breast milk and infant’s feces have been demonstrated [22]. High levels of fecal LF, particularly in preterm infants, may represent an important factor in the initiation, development, and/or composition of the neonatal gut microbiota contributing to the immunologic maturation and well-being of the newborn, especially in preterm infants [22].

Therefore, a mother’s own milk helps to shape developing core gut microbiota that can improve growth and neurodevelopment and decrease the risk of NEC and late-onset sepsis [19,21]. It is for all these reasons that human milk should be the primary enteral diet of premature infants.

##### Type of Delivery

At birth, the intestine is sterile and devoid of bacteria. After birth, a rich and dynamic ecosystem develops from mother’s skin, vaginal and fecal microbiota, and environment microbiota contacts. Microbiota colonization varies according to the type of delivery [23,24,25] (Table 1).

With regard to vaginal delivery, newborns acquire a microbiota composition resembling their mother’s vaginal microbiota. Indeed, an analysis of the meconium of newborn infants [24] revealed a strong correlation between the microbiota of the newborn digestive tract and the microbial communities of the mother’s vagina: *Lactobacillus*, *Prevotella,* and *Sneathia*. Biasucci et al. [25] showed that the microbiota of vaginally delivered infants are also characterized by predominant groups such as *Bifidobacterium longum* and *Bifidobacterium catenulatum*. Other facultative anaerobic species such as *Escherichia coli*, *Staphylococcus*, *Bacteroides fragilis*, and *Streptococcus* colonize the infant gut [26,27,28].

On the contrary, infants born by cesarean section (C-section) acquire bacteria derived from hospital environment and mother’s skin: *Staphylococcus, Corynebacterium, Propionibacterium* spp. [24,29]. *Escherichia, Shigella,* and *Bacteroides* species are underrepresented in infants born by cesarean delivery [29]. The intestinal microbiota of neonates delivered by cesarean delivery are less diverse in terms of bacteria species than the microbiota of vaginally delivered infants [25].

Differences between the microbiota of C-section and vaginally born infants have been also detected in analyses performed at 7 years of age [23]. Cesarean birth has been associated with an increased risk of chronic immune disorders such as asthma, systemic connective tissue disorders, juvenile arthritis, inflammatory bowel disease [30], and obesity [31].

##### Methods of Milk Feeding

Studies have demonstrated that formula-fed infants are more often colonized with *Escherichia coli*, *Bacteroides*, and *Clostridium difficile* compared with breastfed infants [29,32]. Regarding Actinobacteria abundance, *Bifidobacterium* spp. have been associated with breastfeeding and formula milk [33,34]. However, breastfed infants generally harbor a more complex and diverse *Bifidobacterium* microbiota than formula-fed infants [34]. Breastfed infants are provided with microbiota having more than a two-fold increase in the number of *Bifidobacterium* cells compared to formula-fed infants [33]. Breastfed infants have more beneficial gut microbiota, with a higher richness and diversity of *Bifidobacterium* spp. and a lower number of *Clostridium difficile* and *Escherichia coli* than formula-fed infants [32] (Table 1).

*Bifidobacterium* spp. are responsible for the fermentation of galactooligosaccharide (GOS), one of the main components of breast milk, to produce SCFAs [35]. Indeed, *Bifidobacterium* spp. have an enzyme named lacto-N-biosidase, which facilitates the assimilation of GOS [36]. If *Bifidobacterium* quantity increases, the quantity of HMOs in feces decreases causing elevated acetate and lactate concentrations and decreasing pH [37]; an HMO consumption by Bifidobacteria in the infant’s gut may suggest a prebiotic effect of HMOs on gut infant microbiota by selectively stimulating *Bifidobacterium* spp. [37]. Another prebiotic effect of β-palmitate, a natural human milk fatty acid and a component of some infant formulas, on gut infant microbiota was demonstrated by positively influencing *Bifidobacterium* spp. (and *Lactobacillus* spp.) abundance [38].

Furthermore, the maintenance of a healthy and balanced mother’s gut microbiota during pregnancy is also considered as an important factor in positively influencing the milk microbiota composition [39]. Additionally, oral probiotic supplementation in mothers with vaginal delivery may increase breast milk *Bifidobacterium* spp. and *Lactobacilli* spp. abundance [39].

These studies have shown that native core microbiota are shaped during early life: the profile of intestinal microbiota in the full-term, vaginally delivered, and breastfed infant with healthy and balanced mother’s milk microbiota is considered healthy. Researches should seek to demonstrate the role of this native core microbiota composition on gut health and subsequent diseases.

##### Weaning Period

The introduction of solid foods and the termination of milk-feeding/weaning coincide with major gut microbiota changes. The abundance of genera *Bifidobacterium*, *Clostridium coccoides*, and *Bacteroides* are predominant after weaning [40]. The microbiota of breastfed children and the microbiota of children previously fed with formula milk become closer to each other (Table 1).

Dietary habits, infant weaning, and feeding practices become determinants and play a crucial role in gut microbiota variations. The introduction of high-fiber and carbohydrate foods (traditional foods) causes an increase in Firmicutes and *Prevotella*, whereas the introduction of high-fiber and animal protein foods causes an increase in Bacteroidetes [41]. Furthermore, Fallani et al. [40] demonstrated that northern European countries are associated with a higher proportion of Bifidobacteria in the infant gut microbiota, whereas higher levels of *Bacteroides* and *Lactobacilli* characterize southern European countries.

#### 2.1.3. Age

At one year old, a child’s microbiota composition has a characteristic abundance of *Akkermansia muciniphila*, Bacteroides, *Veillonella*, *Clostridium coccoides* spp., and *Clostridium botulinum* spp. [42].

Microbiota diversity increases with age until it becomes a stable adult microbiota composition dominated by three bacterial phyla (Table 1): Firmicutes (*Lachnospiraceae* and *Ruminococcaceae*), Bacteroidetes (*Bacteroidaceae*, *Prevotellaceae*, and *Rikenellaceae*), and Actinobacteria (*Bifidobacteriaceae* and *Coriobacteriaceae*) that are the result of maturation due to the influence of genetics, environment, diet, lifestyle, and gut physiology [42]. At approximately three years of age, a child’s gut microbiota composition and diversity are most like those of adults [43].

With regard to older people over the age of 70, gut microbiota composition can be affected by digestion and nutrient absorption changes and immune activity weakness. Dietary habit changes (more monotonous) may also weaken gut microbiota diversity. A decrease in anaerobic bacteria such as *Bifidobacterium* spp. and an increase in *Clostridium* and Proteobacteria have been observed [44]. Given the role of *Bifidobacterium* spp. in the stimulation of the immune system and metabolic processes, a Bifidobacteria decrease may partially explain low systemic inflammatory status and malnutrition in older adults [45].

#### 2.1.4. Antibiotics

Gut microbiota composition can be more or less affected by antibiotic use. A follow-up study explored the effect of antibiotics with different modes of action on human gut microbiota composition [46] and demonstrated that antibiotic treatments modify the gut microbiota composition with an abundance/appearance of certain species and a decrease/disappearance of other species (Table 1). Broad-spectrum antibiotics lead to an imbalance between Firmicutes and Bacteroidetes. The bacterial diversity decreases and so does the abundance of these bacteria during the treatments. The alteration of microbiome composition depends on the antibiotic class, dose, period of exposure, pharmacological action, and target bacteria [47] (Table 1). Specific properties of antibiotics such as antimicrobial effects or mode of action are powerful forces for the selection of intestinal microbiota and are partially responsible for the shifts in bacterial composition during antibiotic therapy [46].

Each class of antibiotics has different properties and excretion systems, resulting in different patterns of alteration in the microbiome composition [47].

A cultivation-independent survey of gut microbiota composition of three persons before, during, and after two exposures to the same antibiotic (ciprofloxacin) [48] has been conducted. This study revealed a considerable resilience to antibiotic administration but also suggested that, in some cases, the system retains a memory of past disturbance and that, in all cases, repeated disturbance leads to a persistent regime shift [48]. The impact of antibiotic disturbance on the resilience of microbiota during future antibiotic treatments can thus also vary considerably across individuals [49].

### 2.2. Gut Microbiota Variations between Individuals

We highlighted that the gut microbiota composition varies in the same individual and we will see that it also changes between individuals. These inter-individual variations are principally due to enterotypes, body mass index (BMI) level, and external factors such as lifestyle, exercise frequency, ethnicity, and dietary and cultural habits. The microbiota abundance variations between individuals are illustrated in Table 2.

#### 2.2.1. Enterotypes

Gut microbiota of each individual are specifically characterized by clusters of bacteria named enterotypes [13]. Three enterotypes are characterized by three dominant bacteria clusters (Table 2): *Bacteroides* (enterotype I), *Prevotella* (enterotype II), or *Ruminococcus* (enterotype III). Each enterotype harbors different bacteria genera (Table 1). These three enterotypes are not only enumerations of bacteria; they are also specifically regrouped by functions. Indeed, an enterotype is a functional harmonious association of several bacteria species rather than a systematic addition of bacteria species. Every enterotype is not a clear-cut identity such as blood groups; however, enterotypes characterize individuals, remain stable from adulthood, and can be restored if they are modified. Each enterotype with its distinctive clusters of bacteria and respective functional characteristics defines a distinctive way of generating energy from fermentable substrates available in the colon. Indeed, bacteria clusters of enterotype I derive energy primarily from carbohydrates using principally glycolysis and pentose phosphate pathways, whereas bacteria clusters of enterotypes II and III are able to degrade mucin glycoproteins of the gut mucosal layer. Enterotypes seem to be principally defined according to dietary habits. Understanding the origins and functions of enterotypes may improve the knowledge of the relationships between gut microbiota and human health.

#### 2.2.2. Body Mass Index (BMI) Classes

Several studies [50,51] have examined the impact of childhood BMI on gut microbiota composition and demonstrated that children with overweight or normal BMI are provided with a higher microbial diversity than underweight children. Gut microbiota diversity descends stepwise based on BMI class [50,52].

Bervoets et al. [53] demonstrated that the microbiota of obese children are provided with an elevated Firmicutes-to-Bacteroidetes ratio compared to the microbiota of lean children [53]. Furthermore, low relative proportions of *Bifidobacterium vulgatus* and high concentrations of *Lactobacillus* spp. are observed in the obese microbiota [53]. Riva et al. [54] confirmed that obesity is associated with elevated levels of Firmicutes such as *Ruminococcaceae* and depleted levels of Bacteroidetes such as *Bacteroidaceae* and *Bacteroides*. SCFAs are higher in obese children suggesting elevated substrate utilization. These findings suggest that the dysbiosis of gut microbiota may contribute to the pathophysiology of obesity and the increased ratio of Firmicutes to Bacteroidetes is associated with the increased production of SCFAs and energy harvest from colonic fermentation [54].

The underweight BMI level also revealed profound gut microbiota variations. Borgo et al. [55] performed a comprehensive data analysis comparing gut microbiota and anthropometric traits of 15 anorexia nervosa (AN) women and healthy controls. Results showed that AN intestinal microbiota showed a significant increase in *Enterobacteriaceae* and *Methanobrevibacter smithii* compared with healthy controls [55]. On the contrary, the genera *Roseburia*, *Ruminococcus*, and *Clostridium* were depleted in line with the observed reduction in AN of the total SCFAs [55].

BMI levels represent a valid predictive value for gut microbiota dysbiosis. Studies have demonstrated that gut microbiota variations are correlated with increased or depleted production of SCFAs that may respectively contribute to the pathophysiology of obesity or AN. Interventions such as prebiotics and probiotics may be possible solutions to manage both pediatric obesity [50] and AN patients.

#### 2.2.3. Ethnicity, Dietary Habits, and Cultural Habits

While the microbiome of a healthy individual is relatively stable, gut microbial dynamics can certainly be influenced by lifestyle and dietary cultural choices [56]. A study of European children (fed with the Western diet) and Burkina Faso children (assuming a diet rich in millet/sorghum + local vegetables containing very few lipids and animal proteins) [57] revealed that African children’s microbiota have a remarkable abundance of *Prevotella* and *Xylanibacter*. Furthermore, *Shigella* and *Escherichia* are widely under-represented. Another study [58] compared Hadza hunter-gatherers and Italian gut microbiota. At the phylum level, Hadza gut microbiota are largely enriched in Proteobacteria and Spirochaetes, which are extremely rare in the Italian gut microbiota, whereas Actinobacteria, an important subdominant component of the Italian gut microbiota, are almost absent. At the genus level, Hadza gut microbiota are comparatively enriched in *Prevotella*, *Eubacterium*, *Oscillibacter*, *Butyricicoccus*, *Sporobacter*, *Succinivibrio*, and *Treponema* and correspondingly depleted in *Bifidobacterium*, *Bacteroides*, *Blautia*, *Dorea*, unclassified *Lachnospiraceae*, *Roseburia*, *Faecalibacterium*, *Ruminococcus,* and unclassified *Erysipelotrichaceae*. These two studies showed that African gut microbiota have clearly an enterotype *Prevotella* (enterotype II); the African diet rich in millet/sorghum + local vegetables containing very few lipids and animal proteins allows a mucine degradation in synergy with *Desulfovibrionaceae*. European gut microbiota (Western diet) have principally an enterotype *Bacteroides* (enterotype I): the European diet rich in lipids and animal proteins allows energy production from carbohydrates and proteins through fermentation [13]. David et al. [59] compared plant-based diet microbiome (high fibers and low fats and proteins) with animal-based diet microbiome (low fibers and high fats and proteins) and respective changes in microbiome on either diet and demonstrated that a shift toward an animal-based diet increases the abundance of bile-tolerant microorganisms (*Alistipes*, *Bilophila*, and *Bacteroides*) and decreases the levels of Firmicutes that metabolize dietary plant polysaccharides (*Roseburia*, *Eubacterium rectale*, and *Ruminococcus bromii*). Moreover, microbial activity trades off between carbohydrate and protein fermentation when the type of diet respectively changes. This change occurred only one day after the diet reached the distal gut microbiota. David et al. [59] showed that diet alters human microbiota rapidly and reproducibly.

#### 2.2.4. Exercise Frequency

Bai et al. [50] suggested associations of exercise frequency with gut microbiota composition of young children and adolescents. Daily exercise increases gut microbial diversity (Table 2) with a Firmicutes enrichment microbiota: *Clostridiales, Roseburia, Lachnospiraceae*, and *Erysipelotrichaceae* by producing more SCFAs which may increase the expression of tight junction proteins in colon epithelia to heighten the resistance of the intestinal barrier, reduce mucosal permeability, and inhibit inflammatory cytokines [50,60].

Clarke et al. [61] explored exercise and protein consumption for their impact on the gut microbiota of professional athletes from an international rugby union squad. A higher alpha diversity of gut microorganisms representing more than 20 distinct phyla has been demonstrated in athletes compared with high and low BMI controls [61]. Indeed, this study showed that exercise increases gut microbial diversity (Table 2) and protein consumption positively correlates with microbial diversity. These results provide evidence for a beneficial impact of exercise on gut microbiota diversity, but also indicate that the relationship exercise–microbiota diversity is probably related to accompanying dietary extremes [61].

## 3. Gut Microbiota Variations in Health and Disease

Gut microbiota composition is highly variable. The variation itself is considered as physiological in the context of healthy gut microbiota, according to age, ethnicity, lifestyle, and dietary habits. However, these physiological gut microbiota variations have huge implications in intestinal and extra-intestinal disorders. Indeed, dysbiosis is often defined as an alteration of gut microbiota composition and a cause or a consequence of disorders. It is often difficult to ascertain whether the change is beneficial or detrimental. As for the human conditions, changes represent a challenge for a better adaptation in a new context and may potentiate resilience. However, a chronic external stimulation may be stressful and disruptive for an unstructured ecosystem. We will discuss the correlations between several diseases and alterations of gut microbiota composition.

### 3.1. Intestinal Disorders

#### 3.1.1. Irritable Bowel Syndrome

Irritable bowel syndrome (IBS) is one of the most common gastrointestinal (GI) disorders and the bacterial role has been largely investigated [62].

The study [63] of fecal and colonic mucosal biopsy samples from IBS patients and healthy controls demonstrated a significant reduction in the concentration of aerobic bacteria such as the *Lactobacillus* species in fecal samples from IBS patients compared to healthy controls. Another study [64] demonstrated that the microbial communities of IBS patients are enriched in Proteobacteria and Firmicutes but reduced in Actinobacteria and Bacteroidetes compared to controls. In particular, 16S rDNA sequences belonging to the family *Lachnospiraceae* within the phylum Firmicutes are in greater abundance in the IBS clone library [64].

Other studies [62,65] confirmed and revealed some phyla and genera variations in IBS patients compared to healthy controls: an increase in the Firmicutes-to Bacteroidetes-ratio, a decrease in some Firmicutes families (*Lactobacilli*, *Faecalibacterium*) and the Actinobacteria population (Bifidobacteria, *Collinsella*), and an increase in some Firmicutes families (*Veillonella, Streptococci*, *and Ruminococcus* spp.) and in Proteobacteria (*Enterobacteriaceae* spp.). Therefore, these findings demonstrated a loss of microbial richness that may be involved in amino acid synthesis, in the integrity of cellular junctions, and in inflammatory response, suggesting a weakness of the epithelial barrier functions partially explaining IBS symptoms.

#### 3.1.2. Inflammatory Bowel Disease (IBD)

Inflammatory bowel diseases (IBD) gather idiopathic, chronic, and relapsing inflammatory conditions of the gastrointestinal tract including ulcerative colitis (UC) and Crohn’s disease (CRD).

The findings of Frank et al. [66] indicated a diminution of *Lachnospiraceae* and Bacteroidetes microbial populations and an increase in Proteobacteria families in IBD patients compared to control gut microbiota.

Gut microbiota variations appear to be different between UC and CRD. Indeed, a decreased abundance of the butyrate-producing bacteria *Roseburia hominis* and *Faecalibacterium prausnitzii* has been observed in UC patients relative to controls [67], whereas the opposite has been observed in CRD patients, who are provided with increased *Faecalibacterium prausnitzii* levels in addition to a reduced overall diversity [68].

In the case of CRD patients, another study [69] demonstrated a decrease in *Dialister invisus*, an uncharacterized species of *Clostridium* spp., in *Faecalibacterium prausnitzii*, and in *Bifidobacterium adolescentis* and an increase in *Ruminococcus gnavus*.

Sokol et al. [70] confirmed the reduction of *F. prausnitzii* in CRD patients and also demonstrated that an oral administration of live *Faecalibacterium prausnitzii* reduced the severity of colitis and tended to correct the dysbiosis. These results suggest that counterbalancing dysbiosis using *Faecalibacterium prausnitzii* as a probiotic is a promising strategy in CRD treatment [70].

These results do not demonstrate a causal relationship between microbial dysbiosis and IBD pathophysiology, but they rather suggest that gut microbial unbalances are likely to contribute to disease severity.

#### 3.1.3. Celiac Disease

Celiac disease (CD) is a chronic intestinal inflammatory disorder due to an aberrant immune response to dietary gluten proteins in genetically predisposed individuals. 

Gut microbiota variations may play a significant role in the pathogenesis of CD; indeed, dysbiosis is linked with an inflammatory milieu in celiac patients [71]. Celiac disease is caused by the interplay between gluten, genetic factors, and environmental factors such as gut microbiota [72].

Studies [73,74,75,76] have demonstrated that *Bifidobacterium* spp., *Bifidobacterium longum*, *Clostridium histolyticum*, *C. lituseburense,* and *Faecalibacterium prausnitzii* group proportions are less abundant in untreated CD patients than in healthy controls. Indeed, patients with CD show a reduction in beneficial species (*Lactobacillus* and *Bifidobacterium*) and an increase in those potentially pathogenic (*Bacteroides* and *E. coli*) as compared to healthy subjects [74]. The mucosal layer of CD patients fails to stabilize the gut microbiota and fails to prevent the host from the invasion of harmful antigens and pathogens [71]. This dysbiosis is reduced, but may still remain, after a gluten-free diet [71,73].

#### 3.1.4. Colorectal Cancer (CRC)

Colorectal cancer is the third most common cause of cancer mortality in the world [77]. Structural fecal bacterial segregations between CRC patients and healthy volunteers have been demonstrated [78]. The gut microbiota of the CRC patients were enriched in *Bacteroides fragilis*, *Enterococcus*, *Escherichia/Shigella*, *Klebsiella*, *Streptococcus*, and *Peptostreptococcus* and were impoverished in *Roseburia* and other butyrate-producing bacteria of the family *Lachnospiraceae* [78]. The gut microbiota of the healthy volunteers were enriched in *Bacteroides vulgatus* and *Bacteroides uniformis*. Therefore, gut microbiota variations, such as a reduction of butyrate producers and an increase in opportunistic pathogens, constitute a major structural imbalance of gut microbiota in CRC patients.

Shen et al. [79] demonstrated a higher abundance of Proteobacteria and a lower abundance of Bacteroidetes in CRC cases compared to controls. At the genus level, CRC cases showed an increased abundance of *Dorea* spp. and *Faecalibacterium* spp. and lower proportions of *Bacteroides* spp. and *Coprococcus* spp. than controls.

Genomic analysis identified an association of *Fusobacterium* spp. with colorectal cancer [80]; the microbiota of CRC patients were enriched in *Fusobacterium* spp., whereas the Bacteroidetes and Firmicutes phyla were depleted. Furthermore, *Fusobacterium* spp. may contribute to tumorigenesis by an inflammatory-mediated mechanism [80], but the precise role of Fusobacteria in colorectal carcinoma pathogenesis requires further investigation.

All these findings reveal alterations in CRC microbiota that may contribute to the etiology of colorectal cancer. The extension of these findings may lead to strategies able to manipulate microbiota to prevent colorectal cancer as well as to identify individuals at high risk [79].

### 3.2. Extra-Intestinal Disorders

We highlighted that the pivotal relationship between gut microbiota and the host is involved in many intestinal disorders. Recent advances in research have demonstrated that this mutualistic gut microbiota–host relationship appears to play a critical role for many extra-intestinal diseases such as metabolic diseases and neurological disorders with “gut–brain axis” interactions [81], but also for complex multifactorial diseases such as age-related macular degeneration with “gut–retina axis” interactions [82].

#### 3.2.1. Metabolic Disorders

##### Obesity

Animal studies constitute the majority of causative evidence linking changes in microbial composition to obesity while, in humans, data are more variable. In addition, although the majority of mouse gut species are unique, the mouse and human microbiota are similar at the division level, with Firmicutes and Bacteroidetes dominating [83].

The analysis [83] of gut microbiota of genetically obese ob/ob mice and lean mice all fed the same polysaccharide-rich diet revealed a lower relative abundance (50%) of Bacteroidetes in obese ob/ob mice, whereas the Firmicutes are correspondingly higher. Indeed, higher numbers of *Ruminococcaceae* and *Rikenellaceae* have been observed in leptin-resistant obese (leptin-promoting satiety) and diabetic mice (db/db) compared with their lean littermates [84]. In addition, the higher abundance of *Ruminococcaceae* and *Rikenellaceae* in mice fed a high-fat diet is not only dependent on the ingested diet but is also closely linked with obesity and type 2 diabetes [85]. Furthermore, the association between *Desulfovibrionaceae* abundance and obesity and type 2 diabetes has been demonstrated [86,87]. Other studies [50,88] confirmed the role of Proteobacteria in obesity by producing pro-inflammatory molecules such as lipopolysaccharides and helping to harvest energy and increase host fat storage [88].

On the contrary, researchers [86,89] have recently demonstrated that *Akkermansia muciniphila*, a mucin-degrading bacteria living in the mucus layer [90], decreases in both genetically and diet-induced obese mice. 

All these studies highlight the finding that obesity is associated with changes in the composition of gut microbiota including lower species diversity and shifts in the abundance of genes involved in metabolism. These gut microbiota variations affect the microbiome which has an increased capacity to harvest energy from the diet and these changes are transmissible [91]. Gut microbiota can be considered as a contributing factor to the pathophysiology of obesity and may have potential therapeutic implications.

##### Type 2 Diabetes (T2D)

Several studies [92,93] demonstrated that gut microbiota composition is altered in patients suffering from T2D, but it is not clear whether these changes are a cause or simply a consequence of the disorder.

Larsen et al. [92] showed that the proportions of phyla Firmicutes and *Clostridia* are significantly reduced in the T2D sufferers compared to the healthy group. Furthermore, the ratios of Bacteroidetes to Firmicutes, the ratios of *Bacteroides-Prevotella*, and Betaproteobacteria are highly increased in T2D sufferers compared to healthy patients and positively correlated with plasma glucose. 

A metagenome-wide association study of gut microbiota of T2D Chinese sufferers [94] demonstrated that patients with type 2 diabetes were characterized by a decrease in butyrate-producing bacteria such as *Roseburia* spp. and an increase in various opportunistic pathogens (*Clostridium* spp., *Bacteroides caccae*) as well as an enrichment of other microbial functions conferring sulfate reduction (*Desulfovibrionaceae* spp.).

Gut microbiota interact with various host sensing and signaling pathways, leading to a modulation of the endocrine system, immune responses, nervous system activity, and hence, the predisposition to metabolic diseases. Gut microbiota dysbiosis drives and implies new therapeutic strategies for diabetes and related metabolic diseases [95].

#### 3.2.2. Central Nervous System (CNS)-Related Disorders

The brain and the gut are connected via the gut–brain axis with bidirectional interactions between the central nervous system, the enteric nervous system, and the gastrointestinal tract. These bidirectional interactions enable the brain to influence gastrointestinal functions as well as immune functions.

##### Alzheimer’s and Parkinson’s Diseases

Today, over 46 million people live with dementia worldwide [96]. Alzheimer’s disease (AD) and Parkinson’s disease (PD) are considered the most common forms of dementia in the elderly.

Alzheimer’s disease (AD) is a chronic, rapidly progressive neurodegenerative disorder associated with impaired cognition and cerebral accumulation of amyloid-beta peptides. Cattaneo et al. [97] demonstrated that an increase in *Escherichia/Shigella* abundance and a reduction of *E. rectale* abundance are possibly associated with a peripheral inflammatory state in patients with cognitive impairment and brain amyloidosis. Vogt et al. [98] compared gut microbiota composition from patients with and without a diagnosis of AD and revealed a lower microbial diversity compositionally distinct from control-age and sex-matched individuals, with a decrease in Firmicutes and *Bifidobacterium* abundance and an increase in Bacteroidetes abundance in the microbiome of AD participants.

The gut microbiota variations are also involved in the pathogenesis of PD. Hopfner et al. [99] suggested, in agreement with most other studies [100,101,102], an increase in *Lactobacillaceae* abundance in Parkinson patients compared to healthy controls. Moreover, none of the differences in alpha diversity has been observed between PD cases and controls, whereas beta diversity has been revealed for three bacterial families (*Lactobacillaceae*, *Barnesiellaceae*, and *Enterococcaceae*) [99]. PD is associated with changes in gut microbiota and this gut microbiota dysbiosis may be the mechanism of neuroinflammation that leads to PD pathology [99].

##### Hepatic Encephalopathy

Hepatic encephalopathy (HE) is a complication of cirrhosis and is defined as a spectrum of neuropsychiatric abnormalities in patients with liver dysfunctions. Bajaj et al. [103] linked the gut microbiota composition with cognition and inflammation in HE. Fecal microbiota of cirrhotics were significantly higher in *Enterobacteriaceae*, *Alcaligenaceae*, and *Fusobacteriaceae* and lower in *Ruminococcaceae* and *Lachnospiraceae* compared with controls [103]; moreover, altered higher levels of *Veillonellaceae* were found in HE patients compared with cirrhotics without HE. In the cirrhosis group, *Alcaligenaceae* and *Porphyromonadaceae* were positively correlated with cognitive impairment. *Fusobacteriaceae*, *Veillonellaceae*, and *Enterobacteriaceae* were positively and *Ruminococcaceae* was negatively related to inflammation. Thus, cirrhosis, especially when complicated with HE, is associated with significant gut microbiota alterations compared with healthy individuals. Specific bacterial families (*Alcaligenaceae*, *Porphyromonadaceae*, and *Enterobacteriaceae*) are strongly associated with cognition and inflammation in HE [103].

##### Autism Spectrum Disorders

Compared with the gut microbiota of children without ASD, the gut microbiota of children with ASD is less diverse and exhibits lower levels of *Bifidobacterium* and Firmicutes and higher levels of *Lactobacillus*, *Clostridium*, *Bacteroidetes*, *Desulfovibrio*, *Caloramator*, and *Sarcina* [104,105,106,107]. A study by Wang et al. [108] showed a decrease in *Bifidobacterium* spp. and mucolytic bacteria *Akkermansia muciniphila* in the gut microbiota of autism subjects compared to control subjects.

Williams et al. [109] found an increase in the Firmicutes-to-Bacteroidetes ratio and a significant increase in *Sutterella* compared to their abundance in controls. These findings demonstrated that gut microbiota dysbiosis has close relationships with gastrointestinal and behavioral manifestations of autism.

##### Stress

The bidirectional interactions of gut–brain mean that the brain can also alter gut microbiota composition [110]. A model of social disruption among adult mice [111] demonstrated that exposure to stress results in substantial changes in gut microbiota composition: a decrease in *Bacteroides* spp. abundance and an increase in *Clostridium* spp. abundance have been observed in stress-induced mice relative to controls. The mechanisms by which stress influences gut microbiota composition are unclear but include microbial habitat alterations following stress-induced changes in intestinal mobility and mucin secretion [110,112].

Other studies [62] revealed that chronic psychological stress is reflected in the microbiota composition. Indeed, depression due to chronic stressful life events is associated with an increase in *Enterobacteriaceae*, whereas psychological stress is associated with a reduction in *Lactobacilli* spp. and an increase in *Escherichia coli* and *Pseudomonas* spp.

Gut microbiota variations are closely associated with the onset of these neurological disorders, and the gut bacterial communities may be a target for therapeutic interventions.

## 4. Conclusions

Each healthy human is provided with unique gut microbiota. Core native microbiota are shaped in early life (4–36 months) by gut maturation developing from enterotype, birth gestational age, type of delivery, methods of milk feeding, weaning period, lifestyle, and dietary and cultural habits. After a child reaches 2–3 years old, a relative stability in gut microbiota composition has been demonstrated. Richness and diversity of gut microbiota shaped in early life characterize a healthy gut microbiota composition. However, this optimal healthy gut microbiota composition is different for each individual.

Throughout life, the richer and more diverse the microbiota, the better they will withstand external threats. Indeed, gut microbiota represent a changing ecosystem that is severely tested by many factors such as unbalanced diet, stress, antibiotic use, or diseases. A healthy host–microorganism balance must be respected in order to optimally perform metabolic and immune functions and prevent disease development. Indeed, disturbances to the delicate host–microbe relationship may disrupt the development of the immune system, which may in turn result in diseases [113]. Although the role of gut microbiota is still poorly understood, the close association between gut microbiota dysbiosis and intestinal and extra-intestinal disorders has been demonstrated. This is the reason why dysbiosis can be considered as a biomarker of such disorders and that the study of gut microbiota balances shall be one of the priorities for future therapies to prevent and treat diseases.

## Figures and Tables

**Table 1:** Microbiota variations within individuals.

		Gut Microbiota Abundance							Bacteria Diversity	Ref.
		Actinobacteria	Bacteroidetes	Firmicutes	Proteobacteria	Fusobacteria	Verrucomicrobia	Euryarchaeota		
**Anatomical part of gut tract**	**Small intestine**			*Lactobacillus*	*Enterobacteriaceae* *					[14]
	**Colon**		*Bacteroidaceae* * Prevotellaceae * Rikenellaceae *	*Lachnospiraceae* * Ruminococcaceae *							
**Gestational age**	**Preterm birth (<37 weeks of gestation)**	*Bifidobacterium* spp.↓ *Atopobium* spp.↓	Bacteroides *↓ (non-secretor mothers)	Firmicutes *↓ (non-secretor mothers) *Lactobacillus*↑ *Ruminococcus* spp. *Lachnospiraceae* * Peptostreptococcaceae * Clostridiaceae *	*Enterobacteriaceae* *↑ *Enterococcus* spp.↑				↓	[15,16,19]
	**Full-term birth**	*Bifidobacterium* spp.↑	Bacteroidetes *↑	*Ruminococcus* spp. *Lachnospiraceae* * Peptostreptococcaceae * Clostridiaceae *	*Enterobacteriaceae* *				↑	[16,20]
**Type of delivery**	**Vaginal delivery**	*Bifidobacterium* spp.↑ *Bifidobacterium longum*↑ *Bifidobacterium catenulatum*↑	*Prevotella*↑ *Bacteroides fragilis*↑	*Lactobacillus*↑ *Staphylococcus*↑ *Streptococcus* ↑	*Escherichia*↑	*Sneathia*↑			↑	[22,23,24,25,26]
	**C-section**	*Corynebacterium*↑ *Propionibacterium*↑	*Bacteroides* *↓	*Staphylococcus*↑	*Escherichia*↓ *Shigella*↓				↓	[21,22,23,27]
**Methods of (milk) feedings**	**Breast milk**	*Bifidobacterium*↑↑		*Lactobacillus*↑ *Staphylococcus* ↑	*Enterococcus*↑				↑	[27,30,31,32,38]
	**Artificial milk**	*Bifidobacterium*↑	*Bacteroides*↑	*Clostridium*↑ *Clostridium difficile*↑ *Lactobacillus*↑	*Escherichia*↑				↓	[27,30,31,32]
	**Introduction of solid food**	*Bifidobacterium*↑	Bacteroidetes *↑ *Bacteroides*↑	Firmicutes *↑ Lactobacilli↑ *Clostridium coccoides*↑					↑	[36,37]
**Human age**	**Childhood (first year of life)**	*Bifidobacterium*	*Bacteroides*	*Veillonella* *C. coccoides* *C. botulinum*			*Akkermansia muciniphila*		↑	[38,39]
	**2–3 years old to adult**	*Bifidobacteriaceae * * Coriobacteriaceae *	*Bacteroidaceae* * Prevotellaceae * Rikenellaceae *	*Lachnospiraceae* *Ruminococcaceae*	Proteobacteria *	*Fusobacteria* *	*Akkermansia muciniphila*	*Methanobrevibacter smithii*	↑	[38]
	**Over 70**	*Bifidobacteriaceae*↓		*Clostridium* *↓	Proteobacteria *↑				↓	[40]
**Antibiotic treatments**	**Macrolide**	Actinobacteria *↓	*Bacteroides*↑	Firmicutes *↓	Proteobacteria *↑				↓	[43]
	**Clarithromycin**	Actinobacteria *↓	*Bacteroides*↑	Firmicutes *↓	Proteobacteria *↑				↓	
	**Vancomycin**			*Lactobacillus*↓ *Clostridium*↓					↓	
	**Ciprofloxacin**	*Bifidobacterium*↓	*Alistipes*↓ Bacteroides↑	*Faecalibacterium*↓ *Oscillospira*↓ *Ruminococcus*↓ *Dialister*↓					↓	
	**Clindamycin**	*Bifidobacteriaceae*↓ *Lactobacillus*↓							↓	

* Unknown genera.

**Table 2:** Microbiota variations between individuals.

		Gut Microbiota Abundance							Bacteria Diversity	Ref.
		Actinobacteria	Bacteroidetes	Firmicutes	Proteobacteria	Verrucomicrobia	Euryarchaeota	Spirochaetes		
**Enterotype**	**I**	*Slackia*	*Bacteroides* ^(1)^ *Parabacteroides*	*Clostridiales* * Alkaliphilus *Lactobacillus* *Catenibacterium*	*Geobacter*		*Methanobrevibacter smithii*			[13]
	**II**	*Eggerthella*	*Prevotella* ^(1)^	*Veillonella* *Ruminococcaceae* * Holdemania *Peptostreptococcaceae* * Staphylococcus *Leuconostoc*	*Desulfovibrionaceae* * Rhodospirillum *Helicobacter* *Escherichia* *Shigella*	*Akkermansia muciniphila*				
	**III**	*Gordonibacter*	*Sphingobacterium*	*Ruminococcus*^(1)^ *Staphylococcus* *Marvinbryantia* *Symbiobacterium* *Ruminococcaceae* * Dialister		*Akkermansia muciniphila*				
**BMI level (among children and adolescents)**	**Overweight to obese level**	*Bifidobacterium*↑ *B. vulgatus*↓	Bacteroidetes *↓	*Lactobacillus*↑ Firmicutes *↑	Proteobacteria *↑				↓	[46,47,49,50]
	**Underweight level**			*Roseburia* spp.↓ Ruminococcus↓ Clostridium spp.↓	*Enterobacteriaceae* *↑		*Methanobrevibacter smithii*↑		↓	[46,51]
**Exercise frequency**	**High performance sport**		*Bacteroides*↓	*Ruminococcaceae* *↑ *Lactobacillus*↓ *Lactobacillaceae* *↓	*Succinivibrionaceae* *↑	*Akkermansiaceae*↓			↑	[57]
	**Children and adolescents daily exercise**			*Clostridiales*↑ *Roseburia*↑ *Lachnospiraceae*↑ *Erysipelotrichaceae*↑					↑	[46]
**Type of diet, communities, climate, and geographical sites**	**High-fiber diet (African diet)**	*Bifidobacterium*↓	*Prevotella*↑ *Bacteroides*↓	*Eubacterium*↑ *Oscillibacter*↑ *Butyricicoccus*↑ *Sporobacter*↑ *Blautia*↓ *Dorea*↓ *Lachnospiraceae* *↓ *Roseburia*↓ *Faecalibacterium*↓ *Ruminococcus*↓ *Erysipelotrichaceae* *↓	*Succinivibrio*↑			*Treponema*↑	↑	[53,55]
	**High-fat and animal protein diet (Western diet)**	Actinobacteria *↑	*Bacteroides*↑ *Alistipes*↑ *Barnesiella*↑	*Roseburia*↓ *Eubacterium rectale*↓ *Ruminococcus bromii*↓	*Bilophila*↑				↓	

* Unknown genera. ^(1)^ Main genera.
